# Phase II study of everolimus with biomarker exploration in patients with advanced gastric cancer refractory to chemotherapy including fluoropyrimidine and platinum

**DOI:** 10.1038/bjc.2012.47

**Published:** 2012-02-16

**Authors:** D H Yoon, M-H Ryu, Y S Park, H J Lee, C Lee, B-Y Ryoo, J-L Lee, H-M Chang, T W Kim, Y-K Kang

**Affiliations:** 1 Department of Oncology, Asan Medical Center, University of Ulsan College of Medicine, Seoul, Korea; 2 Department of Pathology, Asan Medical Center, University of Ulsan College of Medicine, Seoul, Korea

**Keywords:** everolimus, advanced gastric cancer, biomarker

## Abstract

**Background:**

To evaluate the activity and safety of everolimus and identify potential biomarkers for efficacy of everolimus in patients with advanced gastric cancer (AGC), who failed both fluoropyrimidine and platinum.

**Methods:**

Fifty-four patients received everolimus (10 mg day^−1^). The primary objective was to determine the 4-month progression-free survival (PFS) rate, assumed to be 30%. We additionally investigated the potential biomarkers for everolimus as an exploratory endpoint in those who underwent tumour biopsies.

**Results:**

Two patients (3.7%) achieved partial response and the disease control rate (DCR) was 38.9%. At a median follow-up duration of 8.7 months, the 4-month PFS rate was 18.4%, not fulfilling the primary hypothesis, with a median PFS of 1.7 months and a median overall survival of 8.3 months. The high expression of pS6^Ser240/4^ at baseline was significantly associated with higher DCR (*P*=0.043) and prolonged PFS (*P*=0.001). Grade 1/2 asthenia (96.3%) recorded as the leading toxicity and hyperglycaemia (20.4%) was the most common non-hematological grade 3/4 toxicity. Three patients experienced grade 3/4 pneumonitis. Notably, two experienced treatment-related deaths.

**Conclusion:**

Everolimus is active against a limited number of patients with AGC. pS6^Ser240/4^ may be a potential predictive biomarker for everolimus, which requires validation. Careful monitoring is necessary despite generally favourable toxicity profile.

Despite a worldwide decrease in incidence, gastric cancer remains the fourth most common cancer type and the second most common cause of cancer-related mortality worldwide (Parkin *et al*, 2005; Kamangar *et al*, 2006). Chemotherapy is the standard of care for advanced or recurrent gastric cancer, but median overall survival (OS) is <1 year, with response rates of around 20–40% (Kang *et al*, 2010). Furthermore, current salvage chemotherapy after failure of first-line fluoropyrimidine-based chemotherapy has produced few and short-lived tumour responses, is effective in very limited patient populations, and causes severe toxicity.

The phosphoinositide-3-kinase (PI3K)/mammalian target of rapamycin (mTOR) pathway is frequently dysregulated in many human cancers, and inhibition of the mTOR pathway as a new therapeutic target is an active area of research (Frattini *et al*, 2005; Velho *et al*, 2005). mTOR, a key protein kinase present in all cells, regulates cell growth, proliferation and survival (Frattini *et al*, 2005; Velho *et al*, 2005; Ciuffreda *et al*, 2010). mTOR acts by directly activating ribosomal protein S6 kinase 1 (S6K1) and inhibiting a translational repressor, 4E-binding protein 1 (4EBP1) (Iwenofu *et al*, 2008). S6K1 phosphorylates the S6 protein of the 40s ribosomal subunit at several sites, including Ser 235/236 and Ser 240/244, leading to initiation of protein synthesis (Dufner *et al*, 1999; Iwenofu *et al*, 2008).

Everolimus is a novel macrolide derivative of rapamycin that inhibits the ability of mTOR to phosphorylate S6K1 and 4EBP1, thereby causing G0/G1 arrest and inhibiting cell-cycle progression in cancer cells (Bjornsti and Houghton, 2004). In the first phase II study on patients with advanced gastric cancer (AGC), everolimus was well tolerated with a promising disease control rate (DCR) (Doi *et al*, 2010). Based on these results, a large-scale phase III trial investigating the effects of everolimus on AGC is currently ongoing. However, the molecular determinants that predict the responsiveness of tumour cells to everolimus remain to be established. In the current phase II trial of everolimus involving heavily pretreated AGC patients, we have conducted immunohistochemical (IHC) analysis of molecules related to the mTOR signalling pathway from baseline and/or on-therapy sequential biopsies.

## Patients and methods

### Patients

This is a prospective, open-label, single arm phase II study. Patients with advanced, unresectable and histologically confirmed adenocarcinomas of the stomach were eligible if they met the following inclusion criteria: (1) age 18–75 years, (2) Eastern Cooperative Oncology Group performance status 0–2, (3) prior failure of first-line chemotherapy, including fluoropyrimidine (5-FU) or related drugs (capecitabine, doxifluridine, S1 or tegafur-uracil (UFT)) and platinum (cisplatin or oxaliplatin) chemotherapy, (4) measurable lesions based on the Response Evaluation Criteria in Solid Tumours (RECIST), (5) no previous radiotherapy to >25% of bone marrow, (6) estimated life expectancy of over 3 months, (7) adequate bone marrow, renal and hepatic functions and (8) written informed consent. Patients were excluded in cases of the brain metastases, significant gastrointestinal bleeding or obstruction and serious comorbid conditions or if they lacked the ability to comply with the protocol requirements. This study was approved by the institutional review board of the Asan Medical Center and registered in clinicaltrials.gov. (NCT00729482).

### Treatment and assessment

Everolimus was administered at a dose of 10 mg (two 5 mg tablets) orally every day until progression of disease, unacceptable toxicity, or study discontinuation for any other reason. Each cycle comprised 28 days of treatment. Treatment was reduced to 5 mg day^−1^ and subsequently to 5 mg every other day for specific toxicities. Tumour response was evaluated every two treatment cycles according to RECIST version 1.0. Toxicities were evaluated at day 1 of each cycle according to the National Cancer Institute Common Terminology Criteria for Adverse Events (NCI-CTCAE) scale, version 3.0.

### Statistics

The primary end-point was progression-free survival (PFS). Secondary end-points included response rate, OS and toxicity profiles. Fleming's single-stage one sample design required 48 assessable subjects to determine whether the proportion of patients surviving without progression at 4 months (16 weeks), *P*, was ⩽0.15 or ⩾0.30, with *α* of 0.05 and *β* of 0.2. Assuming a dropout rate of 10%, total accrual of 54 patients was required. Fisher's exact test was used to examine the effects of biomarkers on DCR and the Wilcoxon's signed-rank test was used to analyse the changes from baseline in various biomarkers after treatment with everolimus. Kaplan–Meier estimates were applied to summarise the distribution of time-to-event variables, such as PFS and OS, and the Cox proportional hazards regression model to identify potential prognostic factors or biomarkers for predicting prolonged PFS or OS. The SPSS programme for Windows (SPSS Inc., Chicago, IL, USA) was employed for statistical analysis.

### Biopsy samples

As an exploratory end-point, the influence of biomarkers on efficacy outcome variables was investigated. The objective of this translational research was to identify the profiles of patients that would benefit from everolimus and determine whether the drug effectively inhibits the mTOR pathway in gastric cancer tissue. Fresh tumour biopsies were obtained at screening (before everolimus treatment, *n*=23) and after two cycles of treatment (*n*=19) for consensual patients who did not receive gastrectomy and thus had cancer lesions in the stomach.

### Immunohistochemical staining

Immunohistochemical staining was performed on formalin-fixed, paraffin-embedded tissue sections using the automatic IHC staining device (Benchmark XT, Ventana Medical Systems, Tucson, AZ, USA). Briefly, 4 *μ*m thick whole tissue sections were transferred onto poly-L-lysine-coated adhesive slides and dried at 74°C for 30 min. After standard heat epitope retrieval for 1 h in ethylene diamine tetraacetic acid, pH.8.0, in the autostainer, samples were incubated with antibodies against phosphorylated mTOR (pmTOR, 1 : 200 dilution, clone 49F9, rabbit monoclonal, Cell Signaling Technology, Danvers, MA, USA), pS6K1 (1 : 100 dilution, clone E175, rabbit monoclonal, Epitomics, Burlingame, CA, USA), pS6 at Ser 235/6 (pS6^Ser235/6^, 1 : 150 dilution, clone 91B2, rabbit monoclonal, Cell Signaling Technology), pS6 at Ser 240/4 (pS6^Ser240/4^, 1 : 200 dilution, rabbit polyclonal, Cell Signaling Technology) and p4EBP1 (1 : 400 dilution, clone 236B4, rabbit monoclonal, Cell Signaling Technology). Sections were subsequently incubated with the UltraView Universal DAB kit (Ventana Medical Systems). Slides were counterstained with Harris hematoxylin. Because of insufficient availability of histological sections, immunostaining for p4EBP1 could not be performed in one follow-up biopsy sample.

### Evaluation of IHC staining data

Immunohistochemical results were scored based on the percentage of positive cells. The proportions of positive tumour cells were categorically scored as 0 (0%), 1 (<10%), 2 (10% to 1/3), 3 (1/3 to 2/3) and 4 (⩾2/3). Aberrant expression of pmTOR, pS6K1, pS6^Ser235/6^, pS6^Ser240/4^ and p4EBP1 was assessed from cytoplasmic staining, and tumour cells showing more than weak staining intensity were interpreted as positive. Negative controls were performed by omitting the primary antibodies.

## Results

### Patient characteristics

From July 2008 to February 2010, 54 eligible patients were entered in the study. Patient characteristics are listed in Table 1. In addition to fluoropyrimidine and platinum (FP), 19 patients were refractory to docetaxel and two failed irinotecan, respectively. Twelve patients were diabetic.

### Drug administration

A total of 180 cycles of everolimus were administered for a median of 2 (range, 1–20) cycles per patient. At the time of data cut-off, three patients were on everolimus and the others had discontinued treatment. Disease progression was the primary reason for treatment termination. Specifically, 44 patients discontinued treatment because of disease progression, five owing to toxicity and two of their own will.

### Efficacy

**Figure 1:**
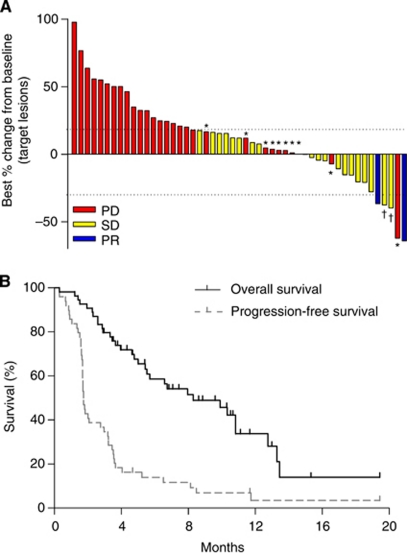
Greatest percentage change from baseline in sum of longest diameters (**A**). Progression-free survival and OS times (**B**). ^*^Progressive disease because of appearance of new metastatic lesions or unequivocal progression of non-target lesions. ^†^Unconfirmed PR. Abbreviations: PD=progressive disease; SD=stable disease.

Responses were not assessable in three patients. One patient refused treatment before response evaluation, another discontinued treatment early because of grade 4 thrombocytopenia and the other died of cardiopulmonary dysfunction before evaluation. Among the 51 assessable patients, two (3.7%) achieved confirmed partial response (PR). One 27-year-old man with multiple liver metastases and abdominal lymph nodes, who had previously failed both S-1 and FOLFOX, attained PR after the eighth cycle and was still on everolimus treatment at the twentieth cycle, with a PR duration of 12.1 months. The other patient, 49-year-old woman with abdominal lymph node metastases, who previously underwent combination chemotherapy of S1 and cisplatin, attained PR after the second cycle and everolimus treatment was maintained for 12 cycles, with a PR duration of 10.0 months at the time of data cut-off. Another 19 patients (35.2%) showed stable disease, resulting in a DCR of 38.9%, whereas decreases in the sizes of target lesions from baseline were observed in 16 patients (29.6%) (Figure 1A). The maximum size reduction rate (64.3%) was observed in the patient achieving confirmed PR. At a median follow-up duration of 8.7 months in surviving patients (range, 3.0–19.4 months), the 4-month PFS rate was 18.4%, with a median PFS of 1.7 months (95% confidence interval (CI) 1.5–2.2 months) (Figure 1B). At the time of data cut-off, 32 patients had died (30 because of disease progression and two from treatment-related causes), resulting in median OS of 8.3 months (95% CI, 4.5–12.1 months).

The PFS and OS values did not change with patient age (<60 years *vs* ⩾60 years), histological differentiation, tumour burden (<6 cm *vs* ⩾6 cm), prior gastrectomy and presence of metastasis in the liver or abdominal lymph node. Peritoneal carcinomatosis and number of metastatic sites (1 *vs* ⩾2) were significantly associated with shorter PFS times in both univariate (hazard ratio (HR) 3.974, 95% CI 1.54–10.23, *P*=0.010; HR 2.319, 95% CI 1.234–4.357, *P*=0.006) and multivariate analyses (*P*=0.043 and 0.017, respectively) (Supplementary Table A1 and A2, online only).

### Biomarker analysis

**Figure 2:**
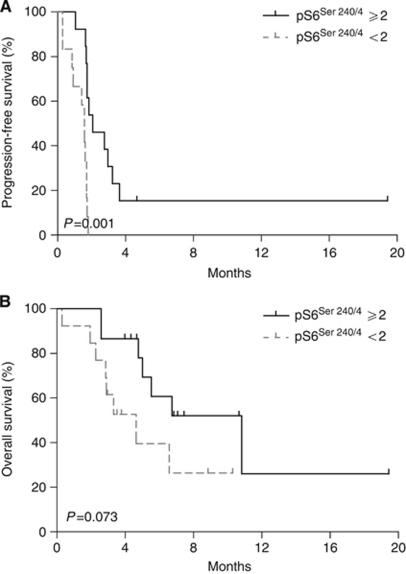
Progression-free survival (**A**) and OS (**B**) times based on baseline expression of pS6^Ser240/4^ (immunohistochemistry score <2 *vs* ⩾2).

**Figure 3:**
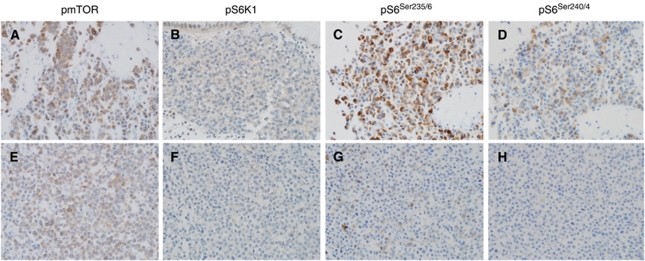
Biomarker expression in the tumour (pmTOR, pS6K1, pS6^Ser235/6^ and pS6^Ser240/4^) at baseline (**A**, **B**, **C** and **D**) and after two cycles of everolimus (**E**, **F**, **G** and **H**) in a selected patient who achieved PR.

In total, 23 baseline biopsies and 19 sequential on-therapy biopsies were available for IHC analysis. Among the IHC biomarkers examined, pS6^Ser235/6^ and pS6^Ser240/4^ were the most frequently expressed (96.4% for both), followed by p4EBP1, pmTOR and pS6K1. Treatment with everolimus resulted in significant inhibition in S6 phosphorylation at Ser 235/6 and Ser 240/4 (*P*=0.0004 and 0.0008, respectively), whereas pS6K1 and p4EBP1 levels were not markedly reduced (*P*=0.2817 and 0.4326) (Supplementary Figure A1, online only, and Figure 3). We explored the potential relationships between baseline or changes in the expression levels of markers and clinical outcomes, including DCR, PFS and OS. High expression of baseline pS6^Ser240/4^ (IHC score ⩾2) or marked decrease (IHC score ⩾2) in pS6^Ser235/6^ expression were significantly associated with higher rates of disease control, achieving clinical response or stable disease (*P*=0.043 and 0.041, respectively) (Supplementary Table A3, online only). In addition, the baseline levels of pmTOR, pS6^Ser235/6^ and pS6^Ser240/4^ were significantly correlated with PFS (*P*=0.005, 0.006 and 0.001, respectively) (Table 2, Figure 2). Additionally, relative decreases in pS6^Ser235/6^ expression by at least 2 IHC scores and pS6^Ser240/4^ by at least 1 IHC score were significant predictors of PFS (*P*=0.005 and 0.031. respectively), whereas the relative increase in pmTOR was associated with prolonged PFS (*P*=0.004). In multivariate analysis with other variables, including the presence of peritoneal carcinomatosis and number of metastatic lesions (1 *vs* ⩾2), baseline expression of pS6^Ser240/4^ (IHC score <2 *vs* ⩾2) was still a significant predictor of PFS (HR 0.246, 95% CI 0.078–0.777, *P*=0.017), whereas baseline and/or changes in expression of pmTOR (<2 *vs* ⩾2) and pS6^Ser235/6^(<3 *vs* ⩾3) showed marginal statistical significance (*P*=0.076 and 0.100) (Supplementary Table A4–A6, online only). Representative biomarker expression levels before and after two cycles of treatment with everolimus from the 27-year-old man showing PR are presented in Figure 3. The second patient achieving PR did not underwent biopsy for biomarker study.

### Toxicity

Treatment was delayed in 24 patients (44.4%). The doses were reduced in five (9.3%) patients because of adverse events, including recurrent grade 2 stomatitis in two patients, recurrent grade 2 thrombocytopenia in two patients and grade 3 thrombocytopenia in one patient. In total, seven patients discontinued treatment because of adverse events or intolerance to everolimus. The mean relative dose intensity per patient was 95.3±10.0%. The most common haematological toxicity was grade 1 or 2 anaemia. Although 15 patients (27.8%) experienced grade 3 or 4 lymphopenia, no clinically significant viral/fungal infection was observed. In addition, no grade 3 or 4 leukopenia or febrile neutropenia was reported (Table 3). In terms of non-hematological grade 3 or 4 toxicities, hyperglycaemia was the most frequent (20.4%), followed by elevation of the *γ*-glutamyl transpeptidase level (18.5%), and electrolyte imbalance, including hyponatremia (16.7%) and hypophosphatemia (16.7%). Six among eleven patients experiencing grade 3 or 4 hyperglycaemia were diabetics on treatment for glycemic control. Three patients experienced grade 3 or 4 pulmonary toxicity. One patient was diagnosed as grade 3 pneumonitis after four cycles of everolimus, which improved following steroid treatment for 2 weeks. However, another patient suffered from diffuse alveolar haemorrhage and died of respiratory failure. In addition, one patient suddenly developed hypoxaemia and heart failure on the eighth day of the first cycle and died of cardiopulmonary dysfunction. The specific cause of death could not be identified, as an autopsy was not performed in this case. In total, two treatment-related deaths were recorded.

## Discussion

In this phase II study, everolimus monotherapy, administered at a dose of 10 mg day^−1^ in unselected patients, failed to achieve a predefined efficacy goal of 4-month PFS rate of 30% against heavily pretreated AGC, despite its mild toxicity. We have further identified potential biomarkers for predicting clinical outcomes following administration of everolimus, based on IHC analysis of baseline and sequential biopsies of primary cancer. Our results suggest that tumours that do not involve activation of the mTOR pathway may not benefit from everolimus treatment.

A DCR of 38.9% with median PFS of 1.7 months and median OS of 8.3 months appeared slightly inferior, compared with the DCR of 56.0%, median PFS of 2.7 months and median OS of 10.1 months reported from the earlier Japanese phase II trial (Doi *et al*, 2010). These discrepancies may be attributed, at least in part, to different patient characteristics, as our trial included very heavily pretreated patients that failed both FP. In addition, 22.1% of patients had peritoneal carcinomatosis, which was a poor prognostic factor in multivariate analysis, compared with 8% of patients in the Japanese trial. Thus, our findings do not necessarily refute the results of the Japanese phase II trial. However, only 2 out of 107 patients from two phase II trials achieved objective response, suggesting that the efficacy of everolimus in the treatment of AGC is unsatisfactory, compared with conventional chemotherapy. A phase II study of docetaxel previously performed in our centre on patients with similar characteristics to those enrolled in the everolimus trial revealed overall response rate of 16.5%, DCR of 57% and median TTP of 2.5 months (Lee *et al*, 2008), which appear superior to data from the present phase II trial. However, two patients treated with everolimus achieved long-term PR that lasted for at least 12.1 and 10.0 months, despite prior failure of S-1 and FOLFOX in one patient, and S1 plus cisplatin in the other. Considering the long-term benefits for these patients, identification of the subpopulation most likely to derive clinical benefit from everolimus should be further explored.

In addition to the presence of peritoneal metastasis and number of metastatic sites, expression of pS6^Ser240/4^ (IHC score <2 *vs* ⩾2), a downstream target of mTOR, at baseline was significantly associated with DCR (8.3% *vs* 46.7%, *P*=0.043) and PFS (median, 2.7 *vs* 1.6 months, *P*=0.001). This finding is consistent with previous results demonstrating positive association of pS6 expression with response to temsirolimus, another agent in the class used to treat renal cell carcinoma (*P*=0.02) (Cho *et al*, 2007). Patients expressing low levels of pS6 did not experience an objective tumour response. pS6 expression has also been linked to sensitivity to the rapalog, AP23573, whereby high pS6 expression is associated with clinical response among metastatic sarcoma patients, but not low expression (Iwenofu *et al*, 2008). We are unsure why expression of pS6K1, a downstream protein of mTOR and an attractive surrogate candidate, did not correlate with the clinical efficacy of everolimus, in contrast to pS6^Ser240/4^. Immunohistochemical evaluation of pS6K1 may be misleading because of its structural similarity to p90 S6 kinase, which is not phosphorylated by mTOR (Castellvi *et al*, 2006) or the amplified signal change through the cascade might only have reached significant level at S6, as it is the substrate of pS6K1, which is the substrate of pmTOR. In addition to pS6^Ser240/4^, high expression of pS6^Ser235/6^ and pmTOR was significantly associated with PFS (*P*=0.006 and 0.005, respectively), although statistical significance in multivariate analysis was not confirmed because of the small sample size. Furthermore, the extent of inhibition of mTOR signalling by everolimus was demonstrated. Specifically, the decrease in pS6^Ser240/4^ and/or pS6^Ser235/5^ expression after treatment with everolimus was significantly associated with prolonged PFS. On the other hand, inhibition of this pathway by everolimus may suppress the negative feedback effect of pS6K1 on an upstream adaptor protein, leading to increased phosphorylation of mTOR, as demonstrated by association of increased pmTOR level with prolonged PFS. We focused on evaluation of downstream substrates of mTOR, which are more likely to reflect the degree of mTOR activation, compared with upstream markers, including the phosphatase and tensin homologue, PI3K and pAkt, which also activate multiple downstream pathways other than mTOR. Nevertheless, our findings do not exclude the possibility that these proteins are potential predictive biomarkers of everolimus. Our findings on potential biomarkers require validation through larger retrospective and prospective trials, as we used a *post-hoc* definition of the criteria for predictive markers of PFS with a small number of samples and smaller number of objective tumour responses. However, these experiments were performed in a subset of the prospective cohort of the clinical trial. Consistently, tumours in which the mTOR pathway was constitutively active and/or signalling inhibited by everolimus were clinically susceptible to the drug, providing proof-of-principle.

Toxicity of everolimus was generally mild, with mostly grade 1 or 2 asthenia recorded as the leading form of toxicity (observed in 96.3% of the patients). The toxicity profile appears consistent with those reported in a large phase III placebo-controlled trial of advanced renal cell carcinoma and the Japanese phase II study (Motzer *et al*, 2008; Doi *et al*, 2010). However, the frequency of grade 3 or 4 hyperglycaemia was slightly higher in the present study, which may be partly attributed to the higher proportion of diabetic patients (22.2%). As described in the study with rapamycin and Akt inhibitor, GSK690693, inhibition of the mTOR pathway may increase insulin resistance and possibly reduce *β*-cell function, necessitating vigilant glucose monitoring and active intervention to control hyperglycaemia (El-Salem *et al*, 2007; Crouthamel *et al*, 2009). Despite a generally favourable safety profile, we experienced two treatment-related mortality cases, one because of diffuse-alveolar haemorrhage and the other of cardiopulmonary dysfunction of unknown cause. In the latter case, the patient died only 8 days after the start of everolimus therapy. Although the direct cause-and-effect relationship is not clear at present, close monitoring of patients is evidently required.

In summary, everolimus monotherapy in unselected patients with AGC does not yield satisfactory therapeutic results. Further investigation is warranted in a subpopulation with tumours in which mTOR signalling is constitutively activated, characterised by high expression of pS6^Ser240/4^. Our results cast a shadow over the ongoing phase III trial of everolimus in unselected AGC patients as salvage therapy. Even in cases of success over placebo, everolimus would have to surpass the effects of conventional chemotherapy regimens, such as docetaxel or irinotecan, to become standard salvage treatment, because of its high cost and relatively-low activity in unselected AGC patients. However, validation of pS6^Ser240/4^ as a potential biomarker and further studies on positive predictive biomarkers for everolimus should uncover the target population susceptible to the drug and establish its role in AGC therapy, considering its long-lasting benefits in a limited number of patients.

## Figures and Tables

**Table 1:** Baseline patient characteristics

**Characteristic**	***N*=54 (%)**
Male (*n*, %)	45 (83.3)
Age, years, median (range)	57.5 (27–72)
	
*ECOG performance*	
0–1	54 (100)
	
*Tumour differentiation*	
Well differentiated	2 (3.7)
Moderately differentiated	27 (50.0)
Poorly differentiated	20 (37.0)
Signet ring cell cancer	5 (9.3)
	
*Site of metastasis*	
Liver	27 (50.0)
Peritoneum	12 (22.2)
Abdominal lymph node	40 (74.1)
Ovary	3 (5.6)
Bone	1 (1.9)
Lung	2 (3.7)
Left supraclavicular lymph node	4 (7.4)
Others^a^	2 (3.7)
Prior gastrectomy	23 (42.6)
Adjuvant chemotherapy	15 (27.7)
	
*Prior chemotherapy*	
Fluoropyrimidine	52 (100)
Platinum	52 (100)
Docetaxel	19 (35.2)
Irinotecan	2 (3.7)

Abbreviation: ECOG=Eastern Cooperative Oncology Group.

a Other site: adrenal gland (*n*=1), psoas muscle (*n*=1).

**Table 2:** IHC markers (at baseline biopsy) *vs* progression-free survival (PFS) and overall survival (OS)

**Biomarkers (before treatment)**	***N***	**Median PFS (95% CI)**	***P*-value**	**Median OS (95% CI)**	***P*-value**
pmTOR			0.005		0.425
<2	15	1.645 (1.421–1.869)		5.526 (3.148–7.905)	
⩾2	13	2.763 (1.142–4.384)		6.743 (1.638–11.849)	
					
*pS6 kinase*			0.150		0.599
<4	16	1.678 (1.484–1.871)		10.822 (NR)	
⩾4	12	1.743 (1.360–2.127)		5.526 (3.094–7.958)	
					
*pS6* ^*Ser 235/6*^			0.006		0.224
<3	14	1.612 (1.491–1.732)		5.526 (2.810–8.243)	
⩾3	14	2.072 (0.566–3.579)		10.822 (4.332–17.313)	
					
*pS6* ^*Ser 240/4*^			0.001		0.073
<2	13	1.579 (1.347–1.811)		4.638 (2.215–7.061)	
⩾2	15	2.763 (1.174–4.352)		10.822 (4.909–16.736)	
					
*p4EBP1*			0.354		0.117
<2	9	1.743 (1.647–1.840)		10.822 (NR)	
⩾2	18	1.743 (1.675–1.811)		5.000 (2.137–7.863)	

Abbreviations: NR=not reached; p4EBP1=phosphorylated 4E binding protein 1; pmTOR=phosphorylated mammalian target of rapamycin.

**Table 3:** Selected drug-related adverse events

	**Grades 1–2, *N* (%)**	***N*=54 Grades >2, *N* (%)**	**Total, *N* (%)**
*Haematologic*			
Leukopenia	16 (29.6)	0 (0.0)	16 (29.6)
Neutropenia	22 (40.7)	1 (1.9)	23 (42.6)
Anaemia	46 (85.2)	5 (9.3)	51 (94.4)
Thrombocytopenia	42 (77.8)	5 (9.3)	47 (87.0)
			
*Non-haematologic*			
Hypercholesterolaemia	24 (44.4)	0 (0.0)	24 (44.4)
Triacylglyceridemia	13 (24.1)	0 (0.0)	13 (24.1)
Hyperglycaemia	36 (66.7)	11 (20.4)	47 (87.0)
Bilirubin	11 (20.4)	3 (5.6)	14 (25.9)
ALP	15 (27.8)	3 (5.6)	18 (33.3)
AST	29 (53.7)	3 (5.6)	32 (59.3)
ALT	18 (33.3)	2 (3.7)	20 (37.0)
*γ*-glutamyl transpeptidase	7 (13.0)	10 (18.5)	17 (31.5)
Hypophosphatemia	5 (9.3)	9 (16.7)	14 (25.9)
Hyponatremia	15 (27.8)	9 (16.7)	24 (44.4)
Asthenia	51 (94.4)	1 (1.9)	52 (96.3)
Myalgia	20 (37.0)	0 (0.0)	20 (37.0)
Diarrhoea	19 (35.2)	0 (0.0)	19 (35.2)
Constipation	15 (27.8)	0 (0.0)	15 (27.8)
Anorexia	43 (79.6)	0 (0.0)	43 (79.6)
Nausea	21 (38.9)	0 (0.0)	21 (38.9)
Vomiting	11 (20.4)	0 (0.0)	11 (20.4)
Abdominal pain	27 (50.0)	1 (1.9)	28 (51.9)
Stomatitis	41 (75.9)	0 (0.0)	41 (75.9)
Skin rash	35 (64.8)	0 (0.0)	35 (64.8)
HFSR	13 (24.1)	0 (0.0)	13 (24.1)
Haemorrhage	17 (31.5)	1 (1.9)	18 (33.3)
Pneumonitis^a^	5 (9.3)	3 (5.6)	8 (14.8)

Abbreviations: ALP=alkaline phosphatase; ALT=alanine aminotransferase; AST=aspartate aminotransferase; HFSR=hand-foot skin reaction.

a Includes interstitial lung disease, lung infiltration, pneumonitis, pulmonary alveolar haemorrhage and other pulmonary toxicities.
