# Supplementary material for: Phase II study of everolimus with biomarker exploration in patients with advanced gastric cancer refractory to chemotherapy including fluoropyrimidine and platinum
Source: Br J Cancer. 2012 Feb 16;106(6):1039–44. doi: 10.1038/bjc.2012.47 (PMC3304416; doi:10.1038/bjc.2012.47)
Supplement: Supplementary Information [file bjc201247x1.doc]

**Appendix**

Table A1. Univariate analysis of progression-free survival (PFS) and overall survival (OS).

| Prognostic factors | N | Median PFS  ( 95% CI) | P-value* | Median OS  ( 95% CI) | P-value* |
| --- | --- | --- | --- | --- | --- |
| Age (years) |  |  | 0.116 |  | 0.250 |
| < 60 | 33 | 1.74 (1.65-1.83) |  | 5.69 (3.92-7.46) |  |
| ≥ 60 | 21 | 3.55 (1.33-5.77) |  | 10.82 (10.08-11.56) |  |
| Tumor differentiation |  |  | 0.641 |  | 0.159 |
| WD or MD | 29 | 1.74 (1.62-1.87) |  | 10.33 (7.98-12.67) |  |
| PD or SRC | 25 | 2.01 (0.41-3.60) |  | 5.43 (3.68-7.18) |  |
| Tumor burden |  |  | 0.693 |  | 0.147 |
| ≥ 6 cm | 27 | 2.01 (1.52-2.50) |  | 12.76 (6.63-13.90) |  |
| < 6 cm | 27 | 1.74 (1.62-1.86) |  | 5.43 (4.11-6.74) |  |
| Prior gastrectomy |  |  | 0.092 |  | 0.208 |
| No | 31 | 1.71 (1.65-1.77) |  | 10.33 (7.36-13.30) |  |
| Yes | 23 | 3.45 (2.96-3.95) |  | 5.53 (3.42-7.63) |  |
| Peritoneal seeding |  |  | 0.010 |  | 0.138 |
| no | 42 | 2.07 (0.35-3.79) |  | 9.90 (6.39-13.42) |  |
| yes | 12 | 1.41 (0.63-2.20) |  | 3.32 (0.00-7.43) |  |
| Liver metastases |  |  | 0.181 |  | 0.798 |
| no | 27 | 3.19 (1.73-4.65) |  | 7.96 (2.48-13.44) |  |
| yes | 27 | 1.71 (1.63-1.79) |  | 8.29 (4.35-12.23) |  |
| Abdominal lymph node |  |  | 0.411 |  | 0.153 |
| no | 14 | 1.68 (1.61-1.75) |  | 12.76 (9.47-16.05) |  |
| yes | 40 | 1.84 (1.49-2.20) |  | 5.69 (3.63-7.75) |  |
| No. of metastases |  |  | 0.006 |  | 0.008 |
| 1 | 24 | 3.52 (3.02-4.02) |  | 12.76 (9.50-16.03) |  |
| ≥ 2 | 30 | 1.74 (1.64-1.84) |  | 4.77 (3.14-6.40) |  |
| Prior chemotherapy |  |  | 0.265 |  | 0.236 |
| FP only | 34 | 2.01 (1.61-2.40) |  | 9.90 (4.85-14.95) |  |
| FP + irinotecan/docetaxel | 20 | 1.68 (1.54-1.82) |  | 5.53 (1.19-9.87) |  |

* P-value from log-rank test

Abbreviations: CI, confidence interval; FP, fluoropyrimidine plus platinum; MD, moderately differentiated; OS, overall survival; PD, poorly differentiated; PFS, progression-free survival; SRC, signet ring cell; WD, well differentiated

Table A2. Multivariate analysis of progression-free survival (PFS) and overall survival (OS)

| Prognostic factors | PFS | | |  | OS | | |
| --- | --- | --- | --- | --- | --- | --- | --- |
|  | P-value* | HR | 95% CI |  | P-value* | HR | 95% CI |
| Peritoneal seeding |  |  |  |  |  |  |  |
| No vs. yes | 0.043 | 2.09 | 1.03-4.24 |  | 0.486 | 1.38 | 0.56-3.40 |
| No of metastases |  |  |  |  |  |  |  |
| 1 vs. ≥2 | 0.017 | 2.18 | 1.15-4.13 |  | 0.023 | 2.46 | 1.13-5.33 |

*P-value from Cox proportional hazards regression model

Table A3. Biomarkers vs. disease control rate

| Prognostic factors | Best response | | P-value |
| --- | --- | --- | --- |
|  | PR or SD | PD |  |
| pmTOR |  |  | 0.087 |
| <2 | 2 (13.3%) | 13 (86.7%) |  |
| ≥2* | 6 (50.0%) | 6 (50.0%) |  |
| pS6^Ser 235/6^ |  |  | 0.103 |
| <3 | 2 (14.3%) | 12 (85.7%) |  |
| ≥3 | 6 (46.2%) | 7 (53.8%) |  |
| pS6^Ser 240/4^ |  |  | 0.043 |
| <2 | 1 (8.3%) | 11 (91.7%) |  |
| ≥2 | 7 (46.7%) | 8 (53.3%) |  |
| Δ pS6^Ser 235/6*^ |  |  | 0.041 |
| <-1 | 5 (62.5%) | 3 (37.5%) |  |
| ≥-1 | 1 (9.1%) | 10 (90.9%) |  |
| Δ pS6^Ser 240/4*^ |  |  | 0.255 |
| <0 | 6 (40.0%) | 9 (60.0%) |  |
| ≥0 | 0 (0.0%) | 4 (100.0%) |  |

*Δ Immunohistochemistry Score (on-therapy score – baseline score)

Abbreviations: PD, progressive disease; PR, partial response; SD, stable disease

Table A4. Multivariate analysis with baseline pmTOR for progression-free survival (PFS) and overall survival (OS).

| Prognostic factors | PFS | | |  | OS | | |
| --- | --- | --- | --- | --- | --- | --- | --- |
| (Baseline) | P-value | HR | 95% CI |  | P-value | HR | 95% CI |
| Cytoplasmic pmTOR (<2 vs. ≥2) | .035 | .338 | .124-0.927 |  | .697 | .795 | .251-2.522 |
| Peritoneal seeding (No vs. Yes) | .192 | 2.011 | .704-5.747 |  | .277 | 2.036 | .565-7.344 |

| Prognostic factors | PFS | | |  | OS | | |
| --- | --- | --- | --- | --- | --- | --- | --- |
|  | P-value | HR | 95% CI |  | P-value | HR | 95% CI |
| Cytoplasmic pmTOR (<2 vs. ≥2) | .033 | .336 | .123-0.918 |  | .906 | .936 | .314-2.793 |
| No. of metastatic sites (1 vs. ≥2) | .422 | 1.640 | .491-5.478 |  | .135 | 4.922 | .608-39.829 |

| Prognostic factors | PFS | | |  | OS | | |
| --- | --- | --- | --- | --- | --- | --- | --- |
|  | P-value | HR | 95% CI |  | P-value | HR | 95% CI |
| Cytoplasmic pmTOR (<2 vs. ≥2) | .076 | .385 | 0.134-1.105 |  | .916 | 1.064 | 0.336-3.367 |
| Peritoneal seeding (No vs. Yes) | .227 | 1.916 | 0.666-5.509 |  | .419 | 1.691 | 0.473-6.409 |
| No. of metastatic sites (1 vs. ≥2) | .505 | 1.520 | 0.444-5.206 |  | .159 | 4.546 | 0.552-37.443 |

Abbreviations: CI, confidence interval; HR, hazard ratio; OS, overall survival; PFS, progression-free survival

Table A5. Multivariate analysis with baseline pS6 Ser 240/4 for progression-free survival (PFS) and overall survival (OS)

| Prognostic factors | PFS | | |  | OS | | |
| --- | --- | --- | --- | --- | --- | --- | --- |
| (Baseline) | P-value | HR | 95% CI |  | P-value | HR | 95% CI |
| pS6 ^Ser 240/4^ (<2 vs. ≥ 2) | .011 | .224 | .071-0.709 |  | .191 | .440 | .128-1.508 |
| Peritoneal seeding (No vs. Yes) | .664 | 1.283 | .416-3.955 |  | .646 | 1.371 | .356-5.285 |

| Prognostic factors | PFS | | |  | OS | | |
| --- | --- | --- | --- | --- | --- | --- | --- |
|  | P-value | HR | 95% CI |  | P-value | HR | 95% CI |
| pS6 ^Ser 240/4^ (<2 vs. ≥ 2) | .005 | .229 | .082-0.644 |  | .199 | .490 | .165-1.455 |
| No. of metastatic sites (1 vs. ≥2) | .309 | 1.827 | .572-5.831 |  | .183 | 4.073 | .515-32.236 |

| Prognostic factors | PFS | | |  | OS | | |
| --- | --- | --- | --- | --- | --- | --- | --- |
|  | P-value | HR | 95% CI |  | P-value | HR | 95% CI |
| pS6 ^Ser 240/4^ (<2 vs. ≥ 2) | .017 | .246 | .078-0.777 |  | .290 | .516 | .151-1.758 |
| Peritoneal seeding (No vs. Yes) | .787 | 1.169 | .376-3.641 |  | .855 | 1.135 | .292-4.416 |
| No. of metastatic sites (1 vs. ≥2) | .332 | 1.789 | .553-5.790 |  | .193 | 3.988 | .496-32.035 |

Abbreviations: CI, confidence interval; HR, hazard ratio; OS, overall survival; PFS, progression-free survival

Table A6. Multivariate analysis with baseline pS6 Ser 235/6 for progression-free survival (PFS) and overall survival (OS)

| Prognostic factors | PFS | | |  | OS | | |
| --- | --- | --- | --- | --- | --- | --- | --- |
| (Baseline) | P-value | HR | 95% CI |  | P-value | HR | 95% CI |
| pS6 ^Ser 235/6^ (<3 vs. ≥ 3) | .051 | .362 | .131-1.003 |  | .467 | .636 | .188-2.152 |
| Peritoneal seeding (No vs. Yes) | .282 | 1.823 | .611-5.346 |  | .408 | 1.758 | .462-6.682 |

| Prognostic factors | PFS | | |  | OS | | |
| --- | --- | --- | --- | --- | --- | --- | --- |
|  | P-value | HR | 95% CI |  | P-value | HR | 95% CI |
| pS6 ^Ser 235/6^ (<3 vs. ≥ 3) | .034 | .352 | .134-0.922 |  | .956 | .967 | .300-3114 |
| No. of metastatic sites (1 vs. ≥2) | .361 | 1.738 | .531-5.689 |  | .120 | 5.043 | .655-38.839 |

| Prognostic factors | PFS | | |  | OS | | |
| --- | --- | --- | --- | --- | --- | --- | --- |
|  | P-value | HR | 95% CI |  | P-value | HR | 95% CI |
| pS6 ^Ser 235/6^ (<3 vs. ≥ 3) | .100 | .415 | .145-1.185 |  | .732 | .808 | .239-2.732 |
| Peritoneal seeding (No vs. Yes) | .330 | 1.727 | .576-5.181 |  | .555 | 1.495 | .394-5.677 |
| No. of metastatic sites (1 vs. ≥2) | .417 | 1.647 | .494-5.496 |  | .180 | 4.199 | .516-34.180 |

Abbreviations: CI, confidence interval; HR, hazard ratio; OS, overall survival; PFS, progression-free survival

Figure A1. Biomarker expression before and after everolimus treatment in patients with assessable paired tumor (p-values from the Wilcoxon-signed-rank test).
